# A Rare Infection of Chromobacterium violaceum in an Immunocompetent Patient: A Case Report

**DOI:** 10.7759/cureus.51148

**Published:** 2023-12-27

**Authors:** Lemis Yavuz, Moza Alhammadi, Rehab Musa, Moataz Hamdi, Mohammed Aldirawi

**Affiliations:** 1 Department of Pediatrics, Al Jalila Children's Speciality Hospital, Dubai, ARE

**Keywords:** contaminated soil, contaminated water, rare infection, cellulitis, chromobacterium violaceum

## Abstract

*Chromobacterium violaceum* is a motile gram-negative bacillus. It lives in water and soil and rarely causes infection in immune-competent patients. It does not respond to the classical treatment and can cause a rapid and progressive illness. Hence, it should be considered in severe infection. Physicians are not generally aware of this organism as a possible cause of infection, and this increases the risk of mortality.

Here, we described a case of a 17-year-old previously healthy girl who had severe necrotic cellulitis that progressed and spread rapidly over a few days despite the treatment with antibiotics. It started after a few days of swimming in a swimming pool. She was treated successfully with a broad spectrum of antibiotics and discharged home.

## Introduction

*Chromobacterium violaceum *is an environmental bacterium that lives in soil and water. It is a rare pathogen of human beings. However, it has a high mortality rate when it does [1]. Usually, the infection starts as a pustule after exposure to a contaminated material, and then it disseminates rapidly. Associated symptoms could be fever, fatigue, vomiting, or any other systemic symptoms [2].

*Chromobacterium violaceum *is a ubiquitous bacteria, so it can be missed and considered a contamination [2]. Hence, physicians should be vigilant about this organism as a rare cause of infection. Morbidity and mortality rates associated with this infection are high due to the aggressive course of illness and not responding to classical antibiotics. So, it is vital to increase awareness about this type of infection [2].

In this case report, we described a previously healthy girl who got necrotizing cellulitis that progressed rapidly in a few days despite the traditional treatment with antibiotics. She was treated successfully with cefepime. 

## Case presentation

**Figure 1:**
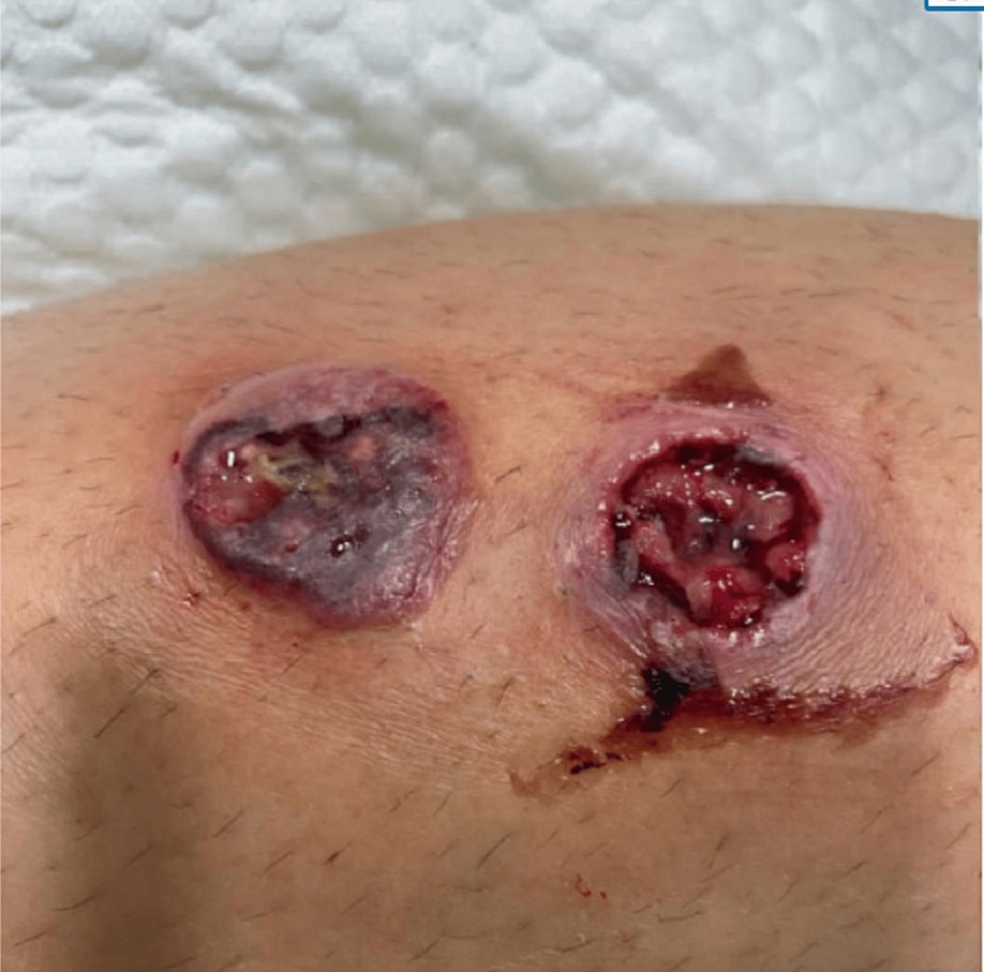
Skin lesions on admission

**Figure 2:**
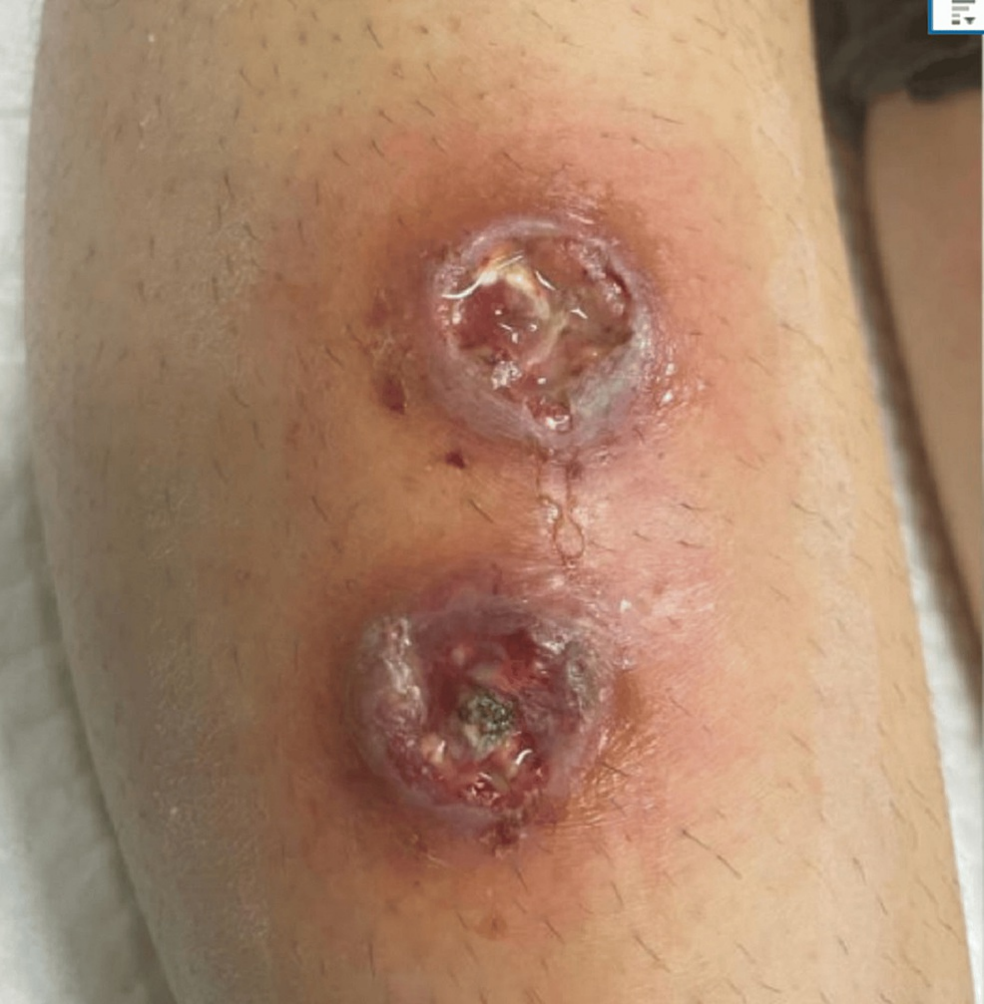
Skin lesions

This is a 17-year-old girl, previously healthy, who was admitted to our facility with ulcers on the right leg. It started as two small pimples four days before the admission. The lesions progressively grew deeper with pus collection. She was treated with fusidic acid cream and amoxicillin-clavulanate acid (1 gram twice daily) orally for four days without improvement. The ulcer worsened, the leg became swollen and red, and then the patient could not walk and started limping. On day three of the illness, new lesions appeared on the right and left legs and started progressing rapidly, with no other associated symptoms (Figures 1, 2).

Two days prior to these symptoms, she was swimming in a home swimming pool with her siblings, and none of them developed similar lesions. Physical examination revealed two necrotic lesions on the right thigh, around 3 to 4 cm, deep into the muscle with pus and blood on the surface. The surrounding skin was red and swollen (Figures 1, 2). Two ulcerative lesions over the right shin with the fascia visible, measuring around 2x2 cm each. Surrounding erythema, warmth, and tenderness (extending downwards over the lateral aspect). The leg was painful to touch, and the patient couldn't bear weight. There were no other findings and the vitals were normal. Blood work upon admission showed normal white blood cells and inflammatory markers. Blood culture and wound culture were sent, and she was started on clindamycin, then changed in 24 hours to cefepime. The wound was cleaned and covered every other day with the Flaminal hydrogel (Flen Health, Esch-sur-Alzette, Luxembourg) and Mepilex AG dressing (Mölnlycke Health Care, Gothenburg, Sweden).

Given the severity of the infection, an MRI of the bone was done to rule out osteomyelitis. Blood culture showed no growth, and wound culture grew *Chromobacterium violaceum* resistant to ceftazidime and sensitive to cefepime. Rheumatology screen and immunochemical work-up were done, and the result was within normal but high IgE. The Whole Exome Sequencing (WES) was sent to look for any underlying immunodeficiency and reported with no significant variation (Table 1).

**Table 1:** Work-up results Findings: Skin and subcutaneous tissue on the lateral aspect of the right leg at the site of skin ulcers showed inflammation and no evidence of BON involvement. MCV - mean corpuscular volume, MCH - mean corpuscular hemoglobin, ANA - antinuclear antibody, WES - whole exome sequencing

Investigation	Results	Reference range
WBC	8.5x10^3^/mcl	4.00-11.00x10^3^
Hgb	12.5 gm/dL	12.0-15.0
MCV	80.3 fL	80.00-100.00
MCH	25.4 pg	27.00-32.00
Platelet	302 000/mcl	150.00-450.00
Neutro absolute	5.6 x10^3^/mcl	2.00-7.00
Lymph absolute	1.7x10^3^/mcl	1.00-4.00
C-reactive protein	4 mg/L	0.0-5.0
Procalcitonin	0.02 ng/ml	<0.5
Erythrocyte sedimentation rate	28 mm/hr	0-10
ANA	Negative	
Double-stranded DNA antibodies	Negative	
Rheumatoid factor	Negative	
HBA1C	4.9 %	4-5.6 %
IgG	14.51 g/l	5.5-16.31
IgE	4910 IU/ML	<100
IgM	1.15 g/l	0.33- 2.93
IgA	1.34 g/l	0.65-4.21
T- and B cells	Normal	
Blood culture	Negative	
Wound culture	Chromobacterium violaceum	Sensitive: cefepime, gentamicin. Resistant: ceftazidime
WES	Uncertain significance in TET2 gene. Variant c 4943 C>T P.(S1648F)	
MRI right leg		

For the first two days after admission, new lesions appeared. However, the patient remained vitally stable, and the ulcers started to improve on day three. After one week, the patient could walk and put weight on her leg. She continued 10 days of cefepime (2g twice daily) in the hospital and then was discharged home on oral ciprofloxacin for four days. Follow-up after two weeks showed significant improvement, and a repeated IgE after six weeks was normal.

## Discussion

The *Chromobacterium violaceum* is a large motile gram-negative bacillus. It is a ubiquitous bacteria that seldom causes any human infection [3]. It was described to affect immunocompromised patients but rarely immunocompetent [2]. Contaminated soil and water are usually the source of infection. The penetration area starts as a small pimple and causes a localized infection, like on the skin or lymph nodes. Then, it can cause bacteremia with necrotizing metastasis, infecting any organ like the liver, brain, or lung [3].

A literature review on this infection was done and showed that males are more susceptible to being infected with a mean age of around 17 years old. The incubation period is about four days, and the course of illness is about 18 days [1]. However, our patient was a female in the same age group. A few other cases have been reported with or without contaminated water or soil exposure [4]. One patient was reported after a fish bite [5]. Similarly, our case started after swimming in a pool. The course of illness differs between cases, from non-specific cellulitis to septic shock and death [5,6]. For that, it is vital to be aware of such infections, and early detection and treatment are essential [1]. Our patient developed severe cellulitis that progressed and spread over a few days despite the treatment with antibiotics. Given the rapid worsening of this infection, the antibiotics were changed to a broad spectrum with pseudomonas coverage. Fortunately, this led to control of the infection. However, new lesions appeared during the first few days of treatment.

The diagnosis can be done through blood or wound culture. As *Chromobacterium violaceum* is an environmental bacterium, it might be missed and considered as contamination. Hence, it is always important to keep this organism in mind and correlate laboratory results with clinical findings [2]. Given the pathology and etiology of this infection, it is recommended to do an immune workup in severe cases. Our patient presented with severe necrotizing cellulitis, so we considered inflammation and infection as a possible cause. For that, a rheumatology screen and immune workup were done. The literature reported that *Chromobacterium violaceum* is more susceptible to ciprofloxacin, gentamicin, and meropenem and is resistant to penicillin and cephalosporine [2]. Our patient did not respond to treatment with amoxicillin-clavulanic acid but responded well to cefepime and oral ciprofloxacin, which she received for 14 days. 

Increased mortality is linked to immune status and antibiotic choice. Hence, it is vital to consider this organism as a possible cause of infection and choose.

## Conclusions

The *Chromobacterium violaceum* is a common environmental organism and can infect immune-compromised patients. It rarely affects immunocompetent. It is a challenging infection as it can easily be missed and considered as contamination, thus increasing comorbidities. The proper choice of antibiotics is vital, and it is recommended to do immune work for patients with severe *Chromobacterium violaceum* infection.
